# Podophyllin and Leptandrin as a Substitute for Mercurials in Diseases of the Digestive Organs

**Published:** 1860-05

**Authors:** 


*On the Use of Podophyllin and Leptandrin as a substitute for Mercurials in diseases of the Digestive Organs.*—Prof. Kirtland of the Cleveland Medical College, remarks in the *Medical Gazette* of March, that "Habit, and occasionally a favorable result, too frequently establish the use of mercurials as a routine, in disorders of the Digestive System, embracing the liver, stomach, alimentary canal, and to some extent the whole glandular structure.

That those preparations are often the most certain and potent means for correcting such disorders, is not to be denied; and I most certainly shall not assume the province of the professional demagogue to decry their use. It should, however, be recollected, that the best of remedies, injudiciously employed, will establish factitious disease, and that very many cases of Hepatic and Digestive derangements, organic and functional, can be traced for their origin to an indiscriminate or too long continued use of mercurials.

Such practice is liable to result in establishing an artificial action, which can only be sustained by a repetition of the same course of means. This artificial condition becomes as imperative in its demands for repetition, as does the abnormal thirst for alcohol, with the inebriate.

For the practitioner to be able to avoid such evils, is a desideratum which I hope and believe to be attainable.

During the last two years, I have in a great measure dispensed with the use of Calomel and Blue Mass in diseases of the aforenamed system, and have substituted therefor a combination of the resinoids of two indigenous plants, *to wit:* Podophyllum Peltatum, and Leptandria Virginica.

The former of these has long been known in popular practice, as the May-Apple er Mandrake, and its medicinal virtues have, perhaps, been more correctly estimated by irregular, than the regular profession.

Standard authors who have noticed it, have copied, one from another, a tissue of errors in regard to its properties, till it is now generally considered to be a mere drastic cathartic, resembling Jalap in its action. Experience has demonstrated to me that in *moderate and suitable* doses it is not drastic, but operates mildly, extensively and equally on the whole alimentary canal. At the same time it is as certain to reach the Liver and Bile-Cyst as is an equivalent dose of calomel, without inducing the sickness and depression which often attend the use of mercurials under such circumstances.

The brief treatise on this vegetable in Wood & Bache's Dispensatory, (8th edition) pages 556, 7 and 8, contains some valuable truths, with at least an equal amount of error. The reputation of the Podyphyllum has been established by its abuse rather than judicious employment; a matter to which I shall again refer.

The latter, the Leptandria Virginica of modern Botanists, was formerly known as the Veronica Virginica, and in domestic medicine as the colored Physic root. I frequently prescribed it, forty years since, and more frequently observed its effects employed as a laxative and cathartic, as it then was extensively used by mothers and nurses, in bowel complaints of children. It seemed in its impression to resemble somewhat Ipecacuanha when administered in small and repeated doses: perhaps less nauseating and diaphoretic, and more laxative.

Both of these plants when prescribed in the form of either decoction or powder of the roots are objectionable, tending to offend the stomach; more from the stimulus of quantity than any medicinal quality. Modern Chemistry and Pharmacy have, however, obviated this objection, by furnishing their active principles in a concentrated form, which can be used in doses so small as not to offend the most sensitive stomach, and at the same time as powerful as the case requires.

My usual prescription for a laxative and aperient as an equivalent for one or two grains of calomel or five grains of blue mass, is the following:

℞ Podophyllin,
Leptandrin, a. a. X grs.
Mix thoroughly—divide into XL powders.

Dose, one powder at bed-time: repeat as occasion may require.

Ale, coffee, or catawba wine forms a convenient and palatable vehicle.

The combination of these two articles was first suggested to me by my friend H. B. Wilcox, M. D., of La Porte Co., Indiana.

It will not be attempted in this communication to specify all the varied morbid conditions of the human system in which the above prescription may be employed, nor the modification and combinations with other agents that may be resorted to by the skilful practitioner to meet individual cases. All this he will readily discern from his knowledge of general principles.

The term *deobstruent*, to designate a class of remedies, is obsolete, yet the above combination of active medicinal principles seems in practice to entitle it to a place under such a head. It is milder, and at the same time more certain to bring into a healthy and active operation every part of the glandular system than any means of my acquaintance. Hence its use is readily suggested in deficient or vitiated secretion of the liver, kidneys and uterus, with their associated morbid conditions.

A caution in regard to the dose of these agents, either single or combined, expeiience shows us to be requisite. On a recent occasion, an intelligent physician was condemning the Podophyllin as a harsh, drastic and irritating cathartic. The query was put to him, " In what dose do you administer it?" The reply was "About two grains; but I do not trouble myself to weigh such articles."

Dr. Zimmerman's Chapter " On False Experience in Medicine," is invaluable. It might be extensively illustrated with instances like the above.

One grain of Opium is a safe narcotic for an adult requiring such an article; but eight grains would destroy the same individual.

One-fourth of a grain of Podophyllin, mixed with an equal amount of Leptandrin, is a full dose for a laxative, but if multiplied by either four or eight, the remedy becomes drastic, harsh and irritating.

Then, again, the practice of portioning out by the eye these potent agents, is unsafe. The eyes, fingers, and judgment of the most experienced may err; but his well-balanced scales, like figures, will not deceive."

